# Converging Effects of Chronic Pain and Binge Alcohol Consumption on Anterior Insular Cortex Neurons Projecting to the Dorsolateral Striatum in Male Mice

**DOI:** 10.1523/JNEUROSCI.1287-23.2024

**Published:** 2024-03-07

**Authors:** Yuexi Yin, David L. Haggerty, Shudi Zhou, Brady K. Atwood, Patrick L. Sheets

**Affiliations:** ^1^ Medical Neurosciences Graduate Program, Indiana University School of Medicine, Indianapolis, Indiana 46202; ^2^ Stark Neurosciences Research Institute, Indiana University School of Medicine, Indianapolis, Indiana 46202; ^3^Department of Pharmacology & Toxicology, Indiana University School of Medicine, Indianapolis, Indiana 46202

**Keywords:** alcohol, insula, mice, pain, slice electrophysiology, striatum

## Abstract

Chronic pain and alcohol use disorder (AUD) are highly comorbid, and patients with chronic pain are more likely to meet the criteria for AUD. Evidence suggests that both conditions alter similar brain pathways, yet this relationship remains poorly understood. Prior work shows that the anterior insular cortex (AIC) is involved in both chronic pain and AUD. However, circuit-specific changes elicited by the combination of pain and alcohol use remain understudied. The goal of this work was to elucidate the converging effects of binge alcohol consumption and chronic pain on AIC neurons that send projections to the dorsolateral striatum (DLS). Here, we used the Drinking-in-the-Dark (DID) paradigm to model binge-like alcohol drinking in mice that underwent spared nerve injury (SNI), after which whole-cell patch-clamp electrophysiological recordings were performed in acute brain slices to measure intrinsic and synaptic properties of AIC→DLS neurons. In male, but not female, mice, we found that SNI mice with no prior alcohol exposure consumed less alcohol compared with sham mice. Electrophysiological analyses showed that AIC→DLS neurons from SNI–alcohol male mice displayed increased neuronal excitability and increased frequency of miniature excitatory postsynaptic currents. However, mice exposed to alcohol prior to SNI consumed similar amounts of alcohol compared with sham mice following SNI. Together, our data suggest that the interaction of chronic pain and alcohol drinking have a direct effect on both intrinsic excitability and synaptic transmission onto AIC→DLS neurons in mice, which may be critical in understanding how chronic pain alters motivated behaviors associated with alcohol.

## Significance Statement

We currently have a poor understanding of how the brain processes the interaction of pain and alcohol. Commonly, pain is associated with increased alcohol consumption. However, our data indicate that nerve injury pain reduces alcohol consumption in mice with no prior exposure to alcohol. Only in these pain–alcohol mice, we found that a specific population of neurons in the anterior insular cortex (AIC) displayed an increase in excitability. Together, this suggests that pain and alcohol interaction can sensitize an AIC circuit that could be targeted for attenuating alcohol intake for treating alcohol use disorders.

## Introduction

Chronic pain is a significant medical problem that affects ∼116 million American adults causing an economic burden of >$635 billion per year (Institute of Medicine, 2011; Egli et al., 2012). Unfortunately, effective treatment of chronic pain remains elusive as current pharmacological strategies often present with negative side effects and strong misuse potential (Jefferies, 2010). Currently, over 25% of people experiencing chronic pain report using alcohol to alleviate suffering (Riley and King, 2009) and those who consume alcohol to cope with pain report greater problematic alcohol use (Zale et al., 2015). Given the impact of both chronic pain and alcohol use disorder (AUD) on public health, an improved understanding of the interaction between these two conditions is critical for improving approaches aimed at prevention and treatment. Previous studies also suggest that the disruptions in the underlying neural circuitry for chronic pain and AUD are similar, yet this connection remains understudied (Watson et al., 1997; Stopponi et al., 2012).

Clinical studies show that the insular cortex (IC) is the most consistently activated region during induced pain experiences in humans (Tracey, 2011; Egli et al., 2012). In rodents, lesioning the IC reduces thermal hyperalgesia and mechanical allodynia (Benison et al., 2011; Coffeen et al., 2011; Zhuo, 2016). The IC is also significantly involved in the alcohol addiction cycle, notably the preoccupation/anticipation stage (Koob and Volkow, 2010; Campbell and Lawrence, 2021). Previous studies demonstrate that local inactivation of the IC disrupts seeking behaviors for alcohol (Ibrahim et al., 2019). However, studies implementing lesion or broad excitation/inhibition of IC do not provide insight into the disruption of specific insular neuron populations that project to distinct down-stream brain regions (Escobar et al., 1998; Jasmin et al., 2003; Benison et al., 2011; Coffeen et al., 2011). Moreover, studies that have investigated changes at the circuit level have focused primarily on IC projections to nucleus accumbens core and amygdala (Seif et al., 2013; Jaramillo et al., 2018b,a; Haaranen et al., 2020).

The anterior insular cortex (AIC), a subregion of IC, is responsible for integrating cognitive and sensory processes such as learning, memory, and sensory perception (Bermudez-Rattoni, 2004; Craig, 2011; Gal-Ben-Ari and Rosenblum, 2011; Zhuo, 2016; Gogolla, 2017). A major target of AIC innervation is the dorsolateral striatum (DLS; Hunnicutt et al., 2016; Munoz et al., 2018; Haggerty et al., 2022). The DLS plays a major role in governing habitual-directed behaviors (Corbit et al., 2012; Alloway et al., 2017; Munoz et al., 2018; Haggerty et al., 2022). Alcohol exposure selectively ablates µ-opioid receptor-mediated long-term synaptic depression at AIC inputs to the DLS (Munoz et al., 2018). Binge-like alcohol drinking produces enhanced ionotropic glutamate receptor-mediated transmission at AIC→DLS synapses that develop over the course of alcohol drinking, but this only occurs in males (Haggerty et al., 2022). However, disruption of AIC→DLS circuits evoked by chronic pain has not been studied. Historically, research in this area focuses on mechanisms of inflammatory and back pain in the ventral, but not dorsal, striatum (Baliki et al., 2012; Martikainen et al., 2015; Dirupo et al., 2021). Given that the DLS is directly connected to the AIC (Gerfen and Bolam, 2016), the AIC→DLS circuitry is of interest in the overlap of alcohol addiction and chronic pain. Here, we investigated the effects of chronic pain on binge-like alcohol drinking behavior and whether the combination of chronic pain and alcohol use alters the intrinsic and synaptic excitability of AIC→DLS neurons.

## Materials and Methods

### Experimental design

#### Animals

Six-week-old male and female C57BL/6J mice were purchased from Jackson Laboratory (JAX #000664) and housed four per cage. Experimental protocols were approved by the Institutional Animal Care and Use Committee from Indiana University School of Medicine (Protocol #22112). Animal welfare guidelines of the National Institutes of Health (NIH, Maryland) were followed. Experiments were performed on 7-week-old animals. After spared nerve injury (SNI) or sham surgeries were performed, mice were switched to individual housing to reduce the possibility of wound reopening and for alcohol drinking experiments.

#### Intracranial injections

Animals were anesthetized with 1.5–2.0% isoflurane in 100% oxygen with a flow rate at 0.8–1.0 L/min. Body temperature was maintained at 37°C using a feedback-controlled heating pad (FHC). Mice were transferred to a stereotaxic frame (Kopf Instruments) and the top of the head area was shaved and disinfected with betadine and isopropyl alcohol. An ∼5 mm incision was made along the midline of the head. A craniotomy was made using a dental drill. Fluorescent red RetroBeads IX (Lumafluor) were injected in the right DLS [coordinates in millimeter relative to bregma: −1.5 A/P, +3.6 M/L, and −3.5 D/V (−40° angle with 15° counterclockwise rotation)] via a Gastight 1701 Hamilton syringe controlled by the UltraMicroPump (World Precision Instruments). Meloxicam (5 mg/ml 10 mg/kg) and buprenorphine (0.03 mg/ml, 0.06 mg/kg) were injected subcutaneously immediately after the surgery. Animals recovered in clean home cages on a heating pad for at least 30 min with *ad libitum* wet feed and water before being transferred back to the vivarium. Animals were monitored 4 d postoperation for signs of wound reopening or excessive pain.

#### SNI model of chronic neuropathic pain

Mouse SNI is a robust long-lasting model for studying chronic neuropathic pain (Bourquin et al., 2006). We chose the SNI model because it provides symptoms similar to clinical neuropathic pain disorders (Cichon et al., 2018), and neuropathic pain is estimated to affect 3–17% of the general population (van Hecke et al., 2014). Mice were anesthetized as described above. The animal's left hind leg was shaved and disinfected with betadine and isopropyl alcohol. An ∼4 mm incision was made through the skin, and the muscle was separated using sterilized wooden dowels exposing the sciatic nerve trifurcation. For SNI surgeries, ∼1 mm of the tibial nerve and the common peroneal nerve were severed, while the sural nerve remained intact. For sham surgeries, the trifurcation was visualized, but not severed. The incision was closed using surgical glue (Vetbond; 3M) and treated with triple antibiotic ointment (Original Strength). Mice recovered and monitored as described in the intracranial injection section.

#### Pain assessment

The pain behavior testing apparatus is an elevated wire-mesh platform, which holds glass cubicles (4 × 4.5 × 4.5 inches) wrapped with black tape. The experimenter was blind to the experimental groups, and animals were allowed to acclimate to the apparatus for 45–60 min. Both hindpaws were tested using von Frey filaments (North Coast Medical) at multiple timepoints: before surgery (baseline), at postoperative day 7 (POD7), and at POD28. The paw withdrawal threshold (PWT) was measured and calculated using the up-down SUDO method (Chaplan et al., 1994; Bonin et al., 2014) to evaluate the development of mechanical allodynia.

#### Drinking-in-the-Dark

The Drinking-in-the-Dark (DID) paradigm is a model of binge-like alcohol drinking behavior in mice (Thiele and Navarro, 2014). Animals were randomly assigned to either alcohol or water groups. Three days after SNI surgeries, mice were transferred into a reversed light/dark cycle room (dark, 06:00–18:00 h; light, 18:00–06:00 h) to acclimate their circadian rhythms for a week (Thiele et al., 2014; Wilcox et al., 2014). Three hours into the dark cycle (09:00 h), water bottles were removed, and tubes containing either 20% alcohol (v/v in water) or water were inserted to the home cages via lickometers constructed as described previously (Godynyuk et al., 2019; Haggerty et al., 2022). Mice had unlimited access to alcohol or water for 2 h/day Monday through Thursday and 4 h on Friday. Four-hour drinking sessions were used on Fridays so mice can achieve blood ethanol concentrations of “binge”-like alcohol drinking. Tubes were weighed before and after each drinking session. Animals were weighed once a week and fluid intake (g/kg) was calculated daily. Mice had abstinence periods during the weekend. This DID cycle was repeated for 3 weeks. Two animals were excluded as they did not cumulatively consume >10 g/kg of alcohol across 3 weeks. In a separate cohort, animals were exposed to saccharin solution: all mice were given 20% alcohol during the first week of DID and then 0.2% saccharin in water (w/v) on the second week. Saccharin concentration was determined based on previous studies (Tampier and Quintanilla, 2009; Bourie et al., 2017; Augier et al., 2018). For animals with alcohol pre-exposure, this 3 week DID paradigm was performed twice—once before SNI/sham surgery and once after.

#### Lickometers

The lickometers have two sides that can hold bottles (Godynyuk et al., 2019). Only one side was used throughout the DID experiment and bottle side was randomized. No side difference was observed, and data were presented collapsed on bottle side. Each drinking behavior was written to device via an infrared beam located directly below the fluid bottle. The data of beam breaks was recorded at minimum every 2 s. Licks are defined as the total number of beam breaks. Lick duration is defined as the duration the beam was broken within one bout. A linear model was fitted between licks and lick duration within each bout for every DID session. Only bouts with residual value between −3 and 3 are included in data analysis (Grecco et al., 2022; Haggerty et al., 2022). This cleaning threshold can exclude inaccurate drinking due to bottle leaking or bottle chewing.

#### Conditioned place preference

Mice underwent a modified conditioned place preference (CPP) test as previously described (Grecco et al., 2022). In summary, mice were allowed to explore both sides of the CPP chamber (Omnitech Electronics) on the pretest day for 20 min, and the side where the animal spent less time was assigned to be the alcohol-paired side. For animals that did not display a strong pretest preference (<60 s), we manually counterbalanced the alcohol-paired side of these mice to different sides of the CPP chamber. Four days of conditioning sessions (5 min) occurred, with saline conditioning (10 ml/kg, i.p.) in the morning (09:00 h) and alcohol (3 mg/kg, i.p.) in the afternoon (13:00 h) to prevent acute alcohol withdrawal during afternoon conditioning sessions. One day after the last conditioning session, the test day occurred where 20 min drug-free exploration of each chamber was assessed. A preference score was calculated as time spent on the saline-paired side subtracted from time spent on the alcohol-paired side during the testing session.

#### Acute brain slice preparation

Brains were dissected and transferred in chilled carbogenated choline (in mM: 25 NaHCO_3_, 1.25 NaH_2_PO_4_, 2.5 KCl, 0.5 CaCl_2_, 7 MgCl_2_, 25 D-glucose, 110 C_5_H_14_CINO, 11.6 C_6_H_7_NaO_6_, 3.09 C_3_H_3_NaO_3_). Coronal brain slices were sectioned in 300 µm using a vibratome (VT1200S Leica). Slices were transferred to carbogenated artificial cerebrospinal fluid (aCSF) solution (in mM: 127 NaCl, 25 NaHCO_3_, 1.25 NaH_2_PO_4_, 2.5 KCl, 25 D-glucose, 2 CaCl_2_, 1 MgCl_2_) at 37°C for 30 min and then at room temperature for at least 45 min prior to recording.

#### Electrophysiology

Recording pipettes were fabricated from borosilicate capillaries using a horizontal puller (P-97; Sutter Instrument) with series resistance between 2 and 4 MΩ. Fluorescently labeled cells were visualized using an LED illumination system (CoolLED pE-4000). For intrinsic excitability recordings, a potassium-based internal solution was used (in mM: 128 K-gluconate, 10 HEPES, 10 sodium phosphocreatine, 4 MgCl_2_, 4 sodium ATP, 0.4 sodium GTP, 3 ascorbic acid, 1 EGTA, 4 mg/ml biocytin). For miniature excitatory postsynaptic current (mEPSC) recordings, a cesium-based internal solution was used (in mM: 128 cesium methanesulfonate, 10 HEPES, 10 sodium phosphocreatine, 4 MgCl_2_, 4 sodium ATP, 0.4 sodium GTP, 3 ascorbic acid, 1 EGTA, 0.5 QX-314). GABAzine (10 µM) and tetrodotoxin (TTX; 0.5 µM) were added to aCSF bath for mEPSC recordings. Whole-cell patch-clamp recordings were performed targeting cells that were 50–80 µm deep in the slice, and the aCSF solution was maintained at 30–31°C. Cells were allowed to stabilize for 5 min before recording. Cells with series resistance higher than 35 MΩ were excluded. Current-clamp recordings were bridge balanced. Recordings were filtered at 4 kHz for intrinsic recordings and at 2 kHz for mEPSC recordings and were digitized at 10 kHz using MultiClamp 700B amplifier (Molecular Devices).

### Statistical analysis

Custom MATLAB (MathWorks) routines were used to process electrophysiology data offline. Statistical analyses were performed with GraphPad Prism 8. DID and lickometer analyses were performed with pingouin (V0.5.3; Vallat, 2018), and data visualization was generated by seaborn (Waskom, 2021) and matplotlib (Hunter, 2007). Differences were considered significant at *p* < 0.05. Results in the text are presented as mean ± SEM. Two-tailed unpaired *t* tests were used to analyze normally distributed data. For data with multiple groups and/or repeated measure, ANOVAs were used with Sidak's post hoc analysis or Tukey's test.

## Results

### SNI induces mechanical allodynia and reduces binge-like alcohol intake in male mice

**Figure 1.**
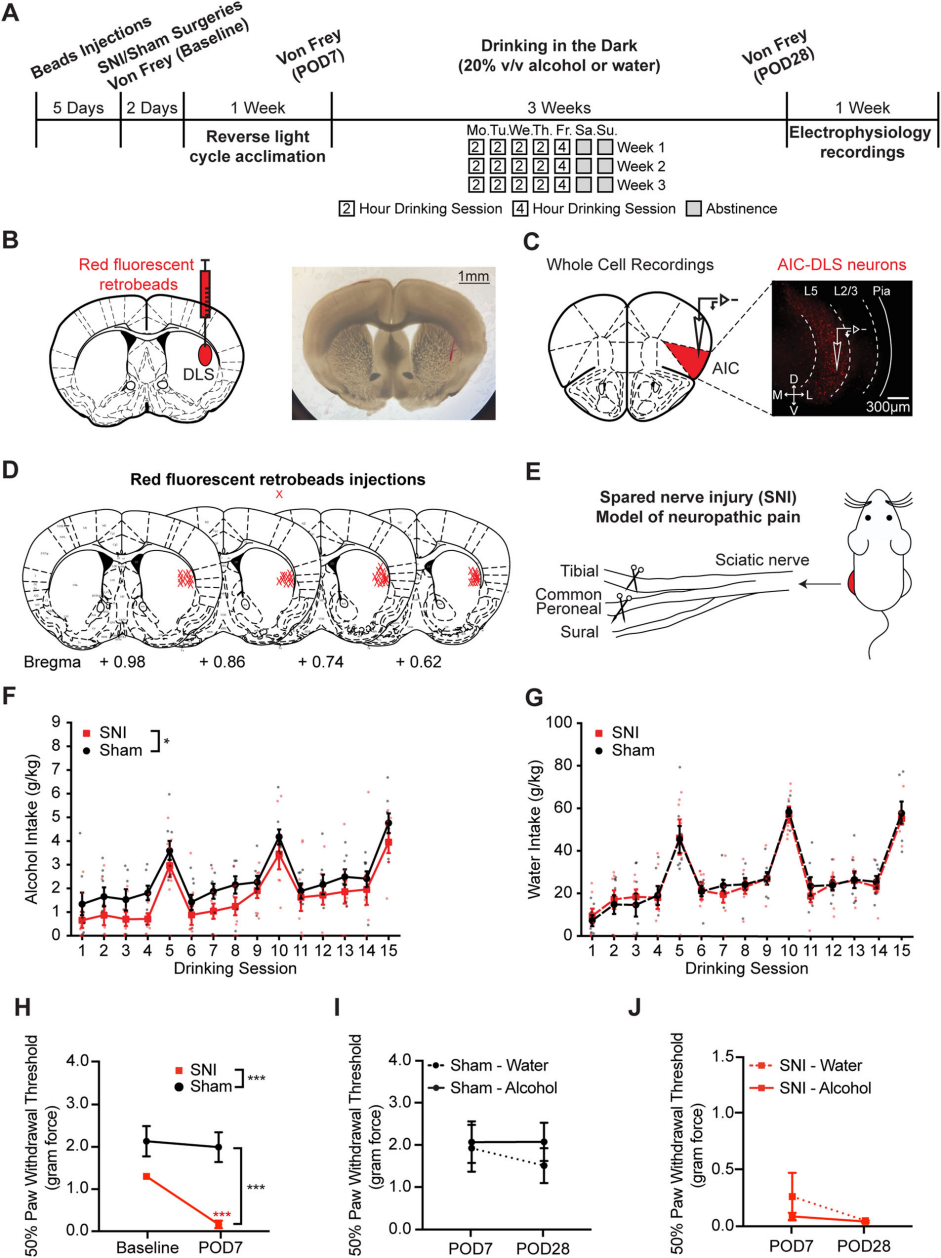
SNI model induces mechanical allodynia and reduces alcohol intake in mice. ***A***, Schematic of the experimental timeline. ***B***, Schematic depicting red retrograde tracer injection into the right DLS and a representative coronal brain section showing red fluorescent beads injection in the right DLS. ***C***, Schematic depicting fluorescently labeled AIC→DLS neurons in the right AIC layer 5 and a representative coronal brain section showing the fluorescently labeled neurons in the right AIC layer 5. ***D***, Locations of retrograde tracer injections. ***E***, Schematic depicting the SNI model of neuropathic pain involving severing the tibial nerve and the common peroneal nerve while keeping the sural nerve intact. ***F***, SNI mice had significantly lower alcohol intake across 15 drinking sessions (sham, *N* = 8; SNI, *N* = 8). Mice were given access to alcohol for 2 h from Monday to Thursday and 4 h on Friday. ***G***, No difference in water intake for sham and alcohol animals across 15 drinking sessions (sham, *N* = 6; SNI, *N* = 6). ***H***, Mechanical allodynia was observed on POD7 after SNI of the sciatic nerve (sham, *N* = 14; SNI, *N* = 14). ***I***, No PWT difference observed from POD7 to POD28 for both water and alcohol drinking sham mice (sham–water, *N* = 6; sham–alcohol, *N* = 8). ***J***, No PWT difference observed from POD7 to POD28 for both water and alcohol drinking SNI mice (SNI–water, *N* = 6; SNI–alcohol, *N* = 8). *p < 0.05. ***p < 0.001. *N*, animal number.

A schematic timeline of the experiments is shown in Figure 1*A*. To identify AIC neurons that project to the DLS, we injected red fluorescent RetroBeads into the right DLS (Fig. 1*B*,*D*). Fluorescently labeled neurons were found primarily in laminar layer 5 (L5) of the right AIC (Fig. 1*C*). Five days following RetroBeads injections, SNI or sham surgery was performed on the left hind leg (Fig. 1*E*). Animals were then acclimated to a reversed light/dark cycle for 7 d (Fig. 1*A*). We found that SNI induced significant mechanical allodynia at POD7 compared with sham surgery (Fig. 1*H*; rmANOVA: surgery, *F*_(1,26)_ = 14.01, *p* < 0.001; time, *F*_(1,26)_ = 67.62, *p* < 0.001; interaction, *F*_(1,26)_ = 41.09, *p* < 0.001; Sidak's post hoc test: POD7 SNI vs sham, *p* < 0.001; SNI baseline vs POD7, *p* < 0.001).

**Figure 2.**
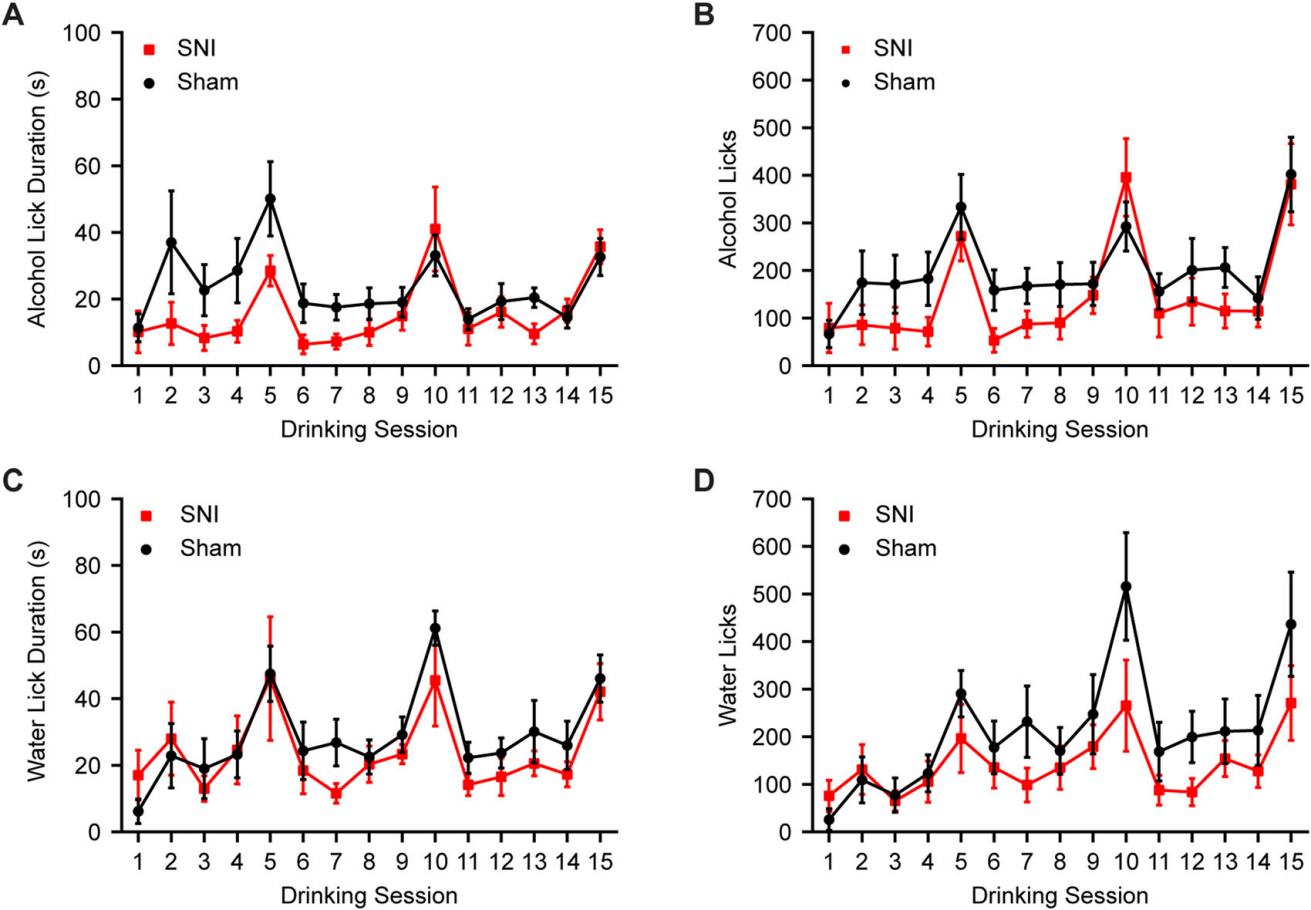
Drinking microstructure analyses of number of licks and lick duration for 3 week DID animals. ***A***, There is a significant interaction of surgery and drinking session on alcohol lick duration. ***B***, SNI and sham group showed no statistical difference in number of alcohol licks. ***C, D***, SNI and sham mice showed no significant differences in (***C***) water lick durations or (***D***) number of water licks (sham–water, *N* = 6; sham–alcohol, *N* = 8; SNI–water, *N* = 6; SNI–alcohol, *N* = 8). *N*, animal number.

Animals subsequently started 3 weeks of DID (Fig. 1*A*). We previously demonstrated that our DID procedure can produce blood alcohol concentrations in excess of 80 mg/dl (Haggerty et al., 2022). We hypothesized that mice with SNI would consume higher amounts of alcohol during DID. Surprisingly, DID data showed that SNI animals consumed significantly less alcohol compared with sham mice across 3 weeks of DID (Fig. 1*F*; rmANOVA: surgery, *F*_(1,14)_ = 7.2206, *p* = 0.0211; time, *F*_(6.139,84.20)_ = 23.1815, *p* < 0.001; interaction, *F*_(14,192)_ = 0.2605, *p* = 0.9968; homoscedasticity test, *p* = 0.159994; no significant difference in Sidak's post hoc analysis). However, no significant difference in water intake was detected between SNI and sham mice (Fig. 1*G*; rmANOVA: surgery, *F*_(1,10)_ = 0.0177, *p* = 0.8962; time, *F*_(3.714,37.14)_ = 38.7673, *p* < 0.001; interaction, *F*_(14,140)_ = 0.2511, *p* = 0.9974). Analyses of drinking microstructure data revealed a significant interaction of lick duration and drinking session between sham and SNI mice in the 3  week alcohol DID (Fig. 2*A*; rmANOVA: surgery, *F*_(1,14)_ = 1.889, *p* = 0.1909; time, *F*_(3.997,53.10)_ = 8.987, *p* < 0.001; interaction, *F*_(14,186)_ = 2.026, *p* = 0.0180). This indicates that lick duration for SNI mice was reduced compared with sham mice in the early stages of 3 week alcohol DID. However, no significant differences were detected in number of licks between sham and SNI mice in the 3 week alcohol DID (Fig. 2*B*; rmANOVA: surgery, *F*_(1,14)_ = 1.253, *p* = 0.2818; time, *F*_(5.694,75.65)_ = 11.71, *p* < 0.001; interaction, *F*_(14,186)_ = 0.8557, *p* = 0.6079). Analyses of water drinking microstructure showed no significant differences between SNI and sham mice for lick duration (Fig. 2*C*; rmANOVA: surgery, *F*_(1,10)_ = 0.6501, *p* = 0.439; time, *F*_(3.260,27.71)_ = 4.353, *p* = 0.011; interaction, *F*_(14,119)_ = 0.7555, *p* = 0.714) or number of licks (Fig. 2*D*; rmANOVA: surgery, *F*_(1,10)_ = 1.306, *p* = 0.280; time, *F*_(2.215,18.83)_ = 4.052, *p* = 0.031; interaction, *F*_(14,119)_ = 0.8524, *p* = 0.611). Overall, this shows that SNI was not inducing a disruption in the animals' ability to consume from bottles with lickometers.

At completion of DID, no changes to the PWT were observed in sham mice (Fig. 1*I*; rmANOVA: fluid, *F*_(1,12)_ = 0.2727, *p* = 0.611; time, *F*_(1,12)_ = 2.080, *p* = 0.175; interaction, *F*_(1,12)_ = 2.167, *p* = 0.167). There is no observed difference between SNI–alcohol and SNI–water animals between POD7 and POD28 either, suggesting that 3 weeks of alcohol exposure did not affect mechanical allodynia (Fig. 1*J*; rmANOVA: fluid, *F*_(1,12)_ = 0.9217, *p* = 0.356; time, *F*_(1,12)_ = 2.107, *p* = 0.172; interaction, *F*_(1,12)_ = 0.8914, *p* = 0.364). Altogether, these data demonstrate that SNI-induced mechanical allodynia persists from POD7 through POD28 across both alcohol and water drinking animals. Overall, data from DID experiments did not support our hypothesis as injured animals (SNI) drank significantly less alcohol than uninjured (sham) controls.

### SNI induces mechanical allodynia but has no effect on DID alcohol consumption in female mice

**Figure 3.**
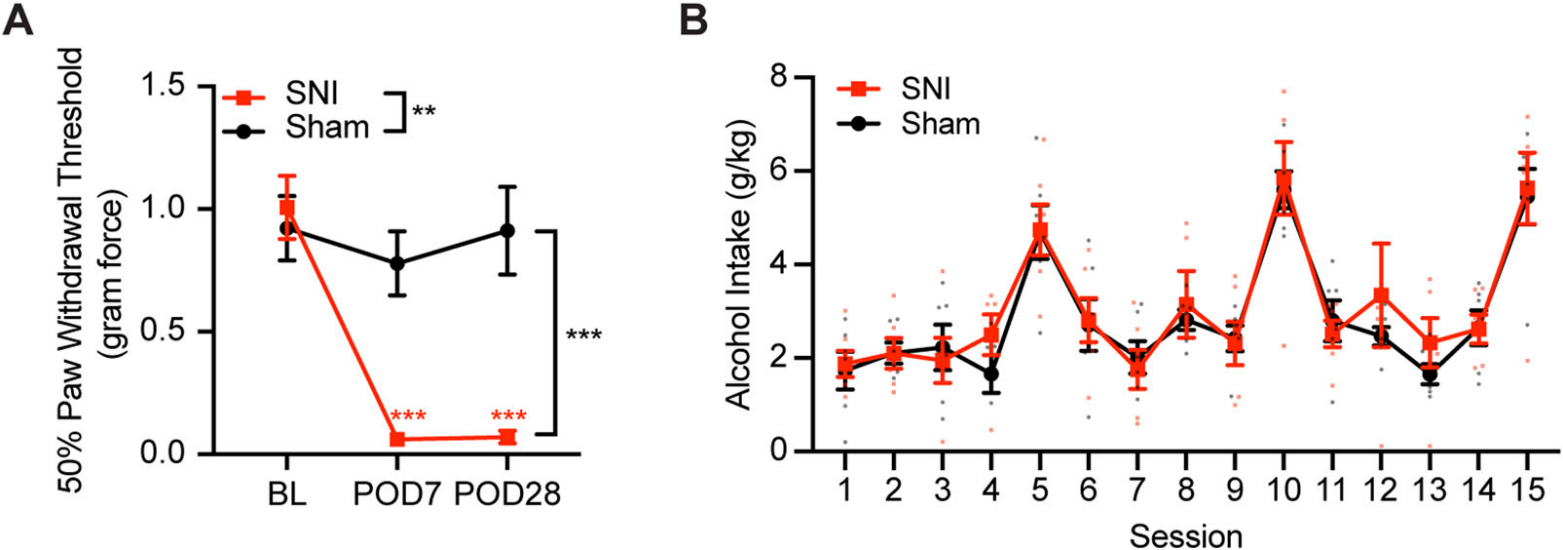
SNI has no effect on DID–alcohol consumption in female mice. ***A***, SNI female mice showed significantly decreased PWT on POD7 and POD28 compared with baseline after surgery. ***B***, SNI and sham female mice had similar alcohol intake (sham, *N *= 6; SNI, *N* = 6). **p < 0.01. ***p < 0.001. *N*, animal number.

We next tested the effects of SNI on DID in female mice. As in male mice, SNI female mice showed significant mechanical allodynia 7 d after surgery, and it persisted until POD28 compared with sham female mice (Fig. 3*A*; rmANOVA: surgery, *F*_(1,10)_ = 15.65, *p* = 0.003; time: *F*_(2,20)_ = 18.61, *p* < 0.001; interaction: *F*_(2,20)_ = 13.43, *p* < 0.001; Sidak's post hoc test: POD7 SNI vs sham, *p* < 0.001; POD28 SNI vs sham, *p* < 0.001; SNI baseline vs POD7, *p* < 0.001; SNI baseline vs POD28, *p* < 0.001; SNI POD7 vs POD28, *p* > 0.999). However, SNI females consumed a similar amount of alcohol compared with sham females across the 3 week DID (Fig. 3*B*; rmANOVA: surgery, *F*_(1,10)_ = 0.1756, *p* = 0.684; time, *F*_(4.310,43.10)_ = 19.05, *p* < 0.001; interaction, *F*_(14,140)_ = 0.3988, *p* = 0.973), which is not what we observed in SNI male mice. While this lack of difference in DID–alcohol consumption between sham and SNI female mice is interesting and needs further investigation, we focused our attention on male mice for this study.

### Both SNI and sham male mice display alcohol placed preference

**Figure 4.**
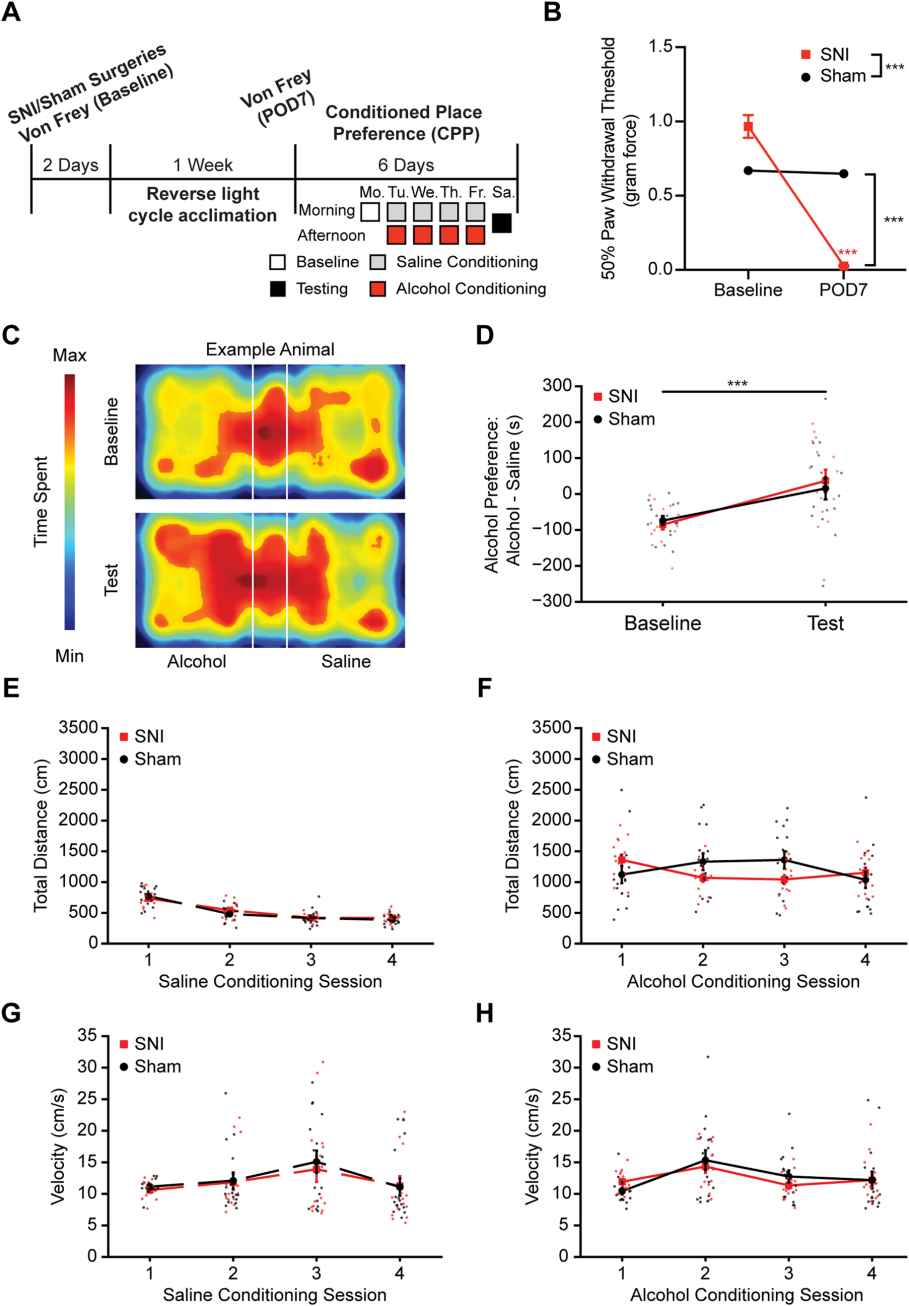
SNI and sham mice both showed alcohol place preference. ***A***, Schematic of the experimental timeline. ***B***, Mechanical allodynia was observed on POD7 after SNI surgery. ***C***, Example animal heatmap for alcohol CPP assay during baseline and test sessions. ***D***, Both sham and SNI mice showed increased alcohol preference during test session compared with baseline session. ***E***, No difference observed in total distance moved during saline conditioning sessions between sham and SNI mice. ***F***, No difference observed in total distance moved during alcohol conditioning sessions between sham and SNI mice. ***G***, No difference observed in velocity during saline conditioning sessions between sham and SNI mice. ***H***, No difference observed in velocity during alcohol conditioning sessions between sham and SNI mice (sham, *N* = 16; SNI, *N* = 15). ***p < 0.001. *N*, animal number.

To examine if reduced alcohol intake in SNI mice was due to a pain-induced reduction in reward value of alcohol, we measured CPP for alcohol reward on a separate cohort of animals (Fig. 4*A*). As in our DID cohort, SNI induced significant mechanical allodynia at POD7 (Fig. 4*B*; rmANOVA: surgery, *F*_(1,29)_ = 17.74, *p* < 0.001; time, *F*_(1,29)_ = 158.1, *p* < 0.001, interaction, *F*_(1,29)_ = 144.6, *p* < 0.001; Sidak's post hoc test: SNI baseline vs POD7, *p* < 0.001; POD7 SNI vs sham, *p* < 0.001). Both sham and SNI mice displayed significantly increased alcohol preference during the test session compared with baseline. However, there was no statistical difference detected between SNI and sham mice's preference scores on test day (Fig. 4*C*,*D*; rmANOVA: surgery, *F*_(1,29)_ = 0.03549, *p* = 0.8519; session, *F*_(1,29)_ = 24.11, *p* < 0.001; interaction, *F*_(1,29)_ = 0.5710, *p* = 0.4560). Total distance traveled and velocity across all saline and alcohol conditioning sessions were not significantly different, indicating no change in locomotive behaviors (Fig. 4*E–H*). These data suggest that reward value of alcohol and alcohol-induced locomotion are similar for both sham and SNI mice and do not contribute to the observed decrease of DID alcohol intake in SNI animals.

### SNI and sham male mice do not display anhedonia-like behaviors

**Figure 5.**
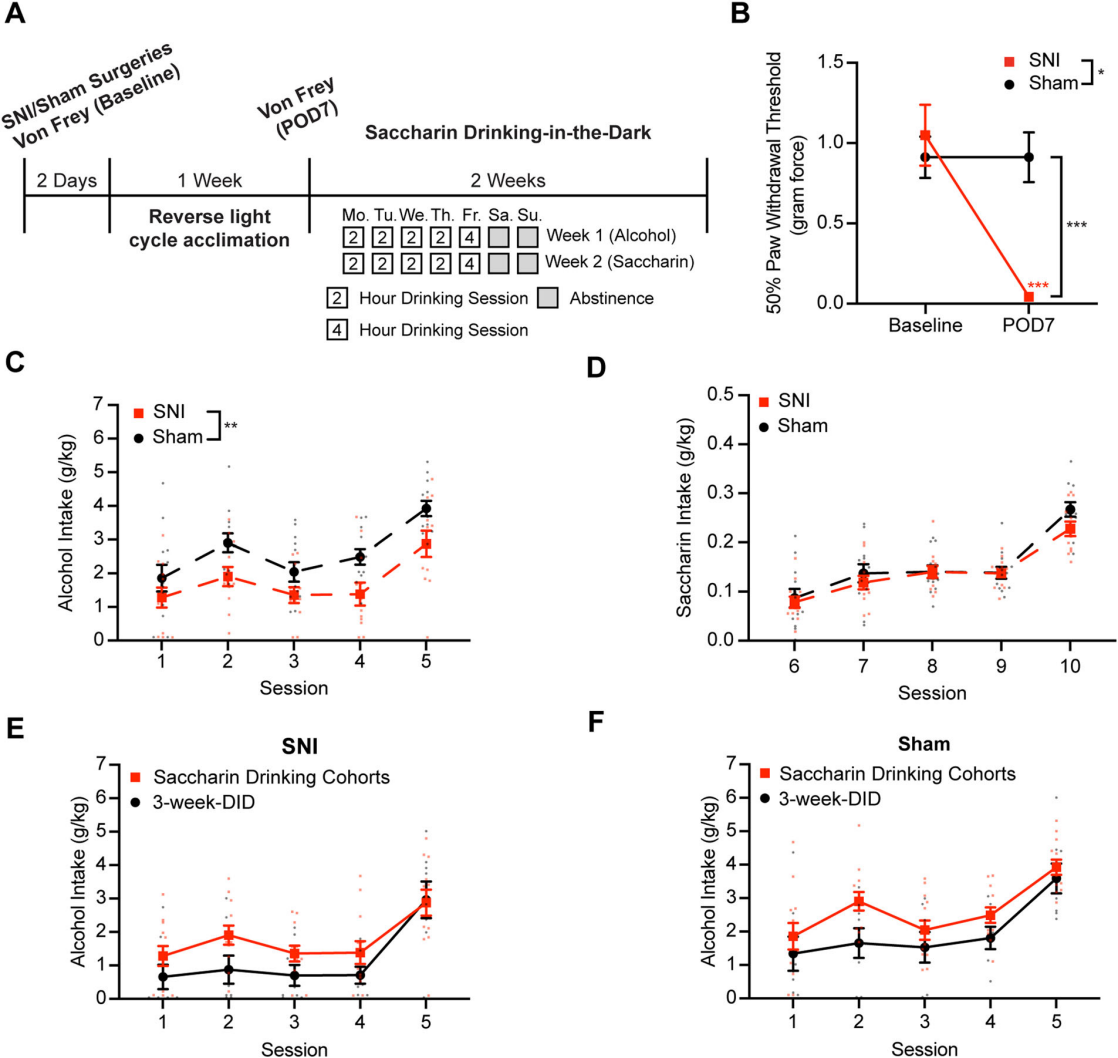
SNI and sham mice had no difference in saccharin intake. ***A***, Schematic of the experimental timeline. ***B***, Mechanical allodynia was observed on POD7 after SNI surgery. ***C***, SNI mice showed lower alcohol intake during the first week of alcohol DID compared with sham mice. ***D***, SNI and sham mice showed similar saccharin intake during the second week of saccharin DID (sham, *N* = 12; SNI, *N* = 12). ***E***, SNI mice in saccharin drinking cohorts and 3 week DID cohorts drank similar amount of alcohol (3 week DID, *N* = 8; saccharin drinking cohorts, *N* = 12). ***F***, Sham mice in saccharin drinking cohorts and 3 week DID cohorts drank similar amount of alcohol (3 week DID, *N* = 8; saccharin drinking cohorts, *N* = 12). *p < 0.05. **p < 0.01. ***p < 0.001. *N*, animal number.

Previous studies suggested chronic pain leads to development of negative emotional states like anhedonia (Garland et al., 2020; Markovic et al., 2021). To test whether reduced alcohol drinking in SNI mice was due to increased anhedonia, we performed a modified DID schedule consisting of a single week of alcohol exposure followed by 1 week of saccharin (0.2% w/v) drinking in SNI and sham mice (Fig. 5*A*). SNI mice showed significant mechanical allodynia 7 d after surgery compared with the baseline (Fig. 5*B*; rmANOVA: surgery, *F*_(1,22)_ = 5.377, *p* = 0.03; time, *F*_(1,22)_ = 19.05, *p* < 0.001; interaction, *F*_(1,22)_ = 19.09, *p* < 0.001; Sidak's post hoc test: SNI baseline vs POD7, *p* < 0.001; POD7 SNI vs sham, *p* < 0.001). During the first week of DID, SNI animals consumed significantly less alcohol compared with sham animals (Fig. 5*C*; rmANOVA: surgery, *F*_(1,22)_ = 9.105, *p* = 0.006; time, *F*_(4,88)_ = 18.07, *p* < 0.001; interaction, *F*_(4,88)_ = 0.4559, *p* = 0.768), consistent with our 3 week DID experiments (Fig. 1*F*). Interestingly, SNI and sham animals consumed the same amount of saccharin following alcohol DID (Fig. 5*D*. rmANOVA: surgery, *F*_(1,22)_ = 0.6946, *p* = 0.414; time, *F*_(2.180,47.95)_ = 91.62, *p* < 0.001; interaction, *F*_(4,88)_ = 1.643, *p* = 0.171). Comparison of baseline alcohol drinking from this saccharin cohort with our 3 week DID animals (Fig. 1*F*) revealed no significant differences (Fig. 5*E*; rmANOVA: cohorts, *F*_(1,18)_ = 2.407, *p* = 0.138; session, *F*_(2.432,43.18)_ = 16.33, *p* < 0.001; interaction, *F*_(4,71)_ = 1.009, *p* = 0.409; Fig. 5*F*; rmANOVA: cohorts, *F*_(1,18)_ = 2.648, *p* = 0.121; session, *F*_(2.949,51.60)_ = 19.82, *p* < 0.001; interaction, *F*_(4,70)_ = 0.9534, *p* = 0.439). These data indicate that reduced DID alcohol consumption in SNI animals is not driven by anhedonia (Verharen et al., 2023).

### SNI in combination with alcohol increases intrinsic excitability of AIC→DLS neurons

**Figure 6.**
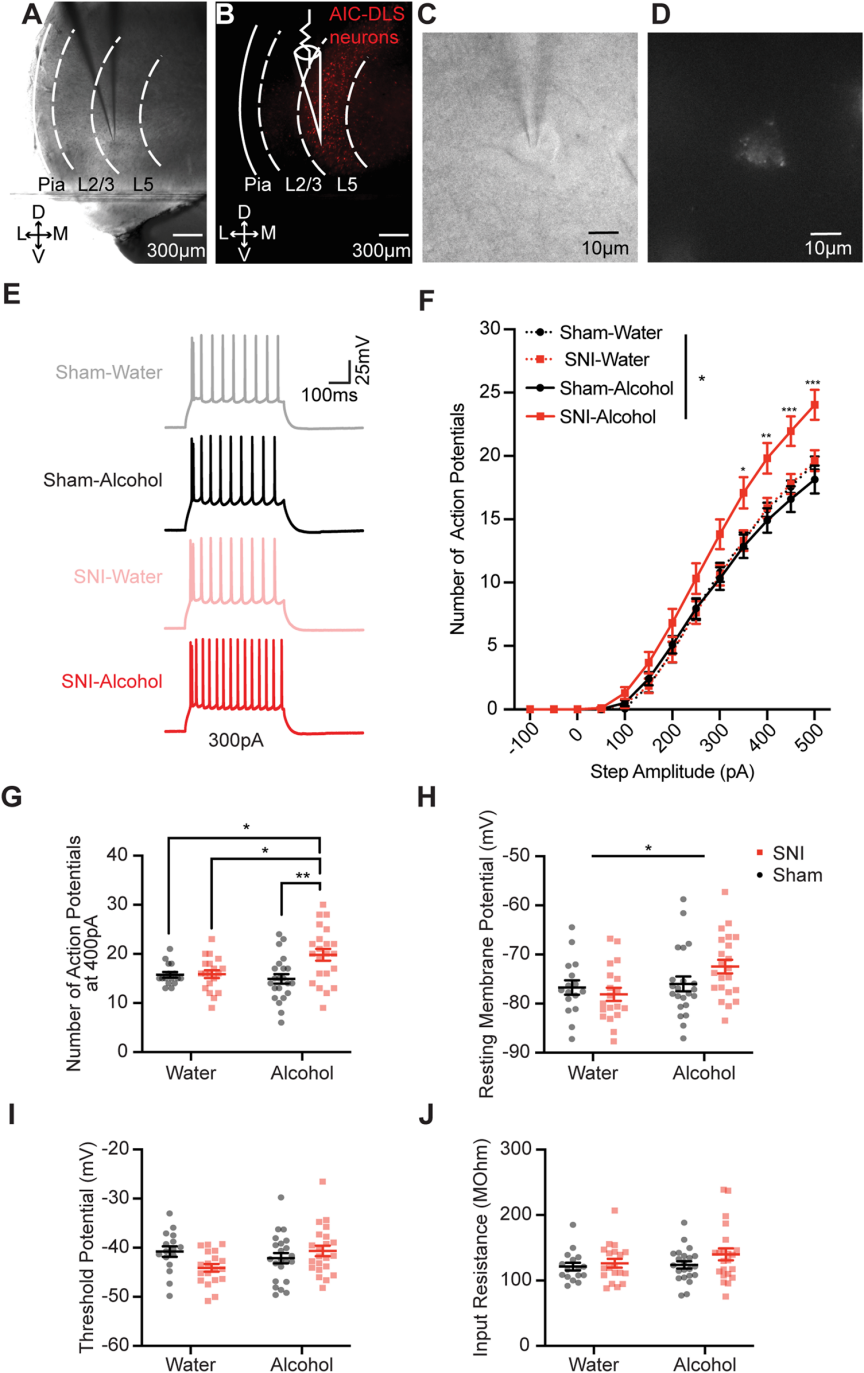
The combination of SNI and alcohol consumption increases the intrinsic excitability of AIC→DLS neurons. Representative images showing fluorescent retrograde labeling of an AIC→DLS neuron recorded in AIC at 4× under (***A***) bright view and (***B***) fluorescent view; at 60× under (***C***) bright view and (***D***) fluorescent view. ***E***, Example traces of current-evoked action potential firing from AIC→DLS neurons from sham–water (gray), sham–alcohol (black), SNI–water (orange), and SNI–alcohol (red) animals. ***F***, Frequency–input (FI) current curves showing that the frequency of action potential firing of AIC→DLS neurons in response to depolarizing current steps in SNI–alcohol mice (*N* = 6, *n* = 22) is significantly higher compared with neurons from sham–water (*N* = 6, *n* = 16), SNI–water (*N* = 5, *n* = 19), and sham–alcohol (*N* = 7, *n* = 22) mice (asterisks represent significant difference between cells from sham–alcohol and SNI–alcohol group). ***G***, AIC→DLS neurons from SNI–alcohol mice had more number of action potentials after injecting 400 pA current compared with neurons from all other groups. ***H***, Alcohol drinking mice displayed a depolarized resting membrane potential compared with water drinking mice. There was no difference in (***I***) threshold potential or (***J***) input resistance. *p < 0.05. **p < 0.01. ***p < 0.001. *N*, animal number; *n*, cell number.

After animals finished 3 weeks of DID, we performed whole-cell electrophysiology recordings on retrogradely labeled L5 AIC→DLS neurons in acute brain slices (bregma 2.46–1.98 mm; Fig. 6*A–D*). Recording analyses revealed a drinking and pain interaction, indicating that AIC→DLS neurons from SNI–alcohol mice displayed an increased frequency of action potentials in response to depolarizing current steps compared with all other mice (Fig. 6*E*,*F*; rmANOVA: current, *F*_(12,900)_ = 902.3, *p* < 0.001; fluid, *F*_(1,75)_ = 3.016, *p* = 0.087; surgery, *F*_(1,75)_ = 4.563, *p* = 0.036; current × fluid, *F*_(12,900)_ = 1.829, *p* = 0.040; current × surgery, *F*_(12,900)_ = 4.713, *p* < 0.001; fluid × surgery, *F*_(1,75)_ = 4.302, *p* = 0.042; current × fluid × surgery, *F*_(12,900)_ = 4.421, *p* < 0.001). Analysis of action potentials evoked by a 400 pA step-current injection revealed that AIC→DLS neurons from SNI–alcohol mice fired significantly more action potentials compared with neurons from all other groups (Fig. 6*G*; two-way ANOVA: fluid, *F*_(1,75)_ = 2.467, *p* = 0.120; surgery, *F*_(1,75)_ = 6.632, *p* = 0.012; interaction, *F*_(1,75)_ = 5.894, *p* = 0.018; Tukey's test: sham–water vs SNI–alcohol, *p* = 0.027; SNI–water vs SNI–alcohol, *p* = 0.025; sham–alcohol vs SNI–alcohol, *p* = 0.002; Table 1). AIC→DLS neurons from alcohol-DID mice displayed a significantly depolarized resting membrane potential compared with mice that consumed water, but no statistical difference between SNI–alcohol and sham–alcohol AIC→DLS neurons was detected (Fig. 6*H*; two-way ANOVA: fluid, *F*_(1,75)_ = 4.900, *p* = 0.030; surgery, *F*_(1,75)_ = 0.5395, *p* = 0.465; interaction, *F*_(1,75)_ = 2.902, *p* = 0.093; Table 1). No significant main differences were detected in action potential threshold potential or input resistance across the four experimental groups (Fig. 6*I*,*J*; Table 1). Together, these results suggest that the interaction of SNI and alcohol consumption increases intrinsic excitability of AIC→DLS neurons.

**Table 1.** Subthreshold and firing properties of AIC→DLS neurons from sham–water, SNI–water, sham–alcohol, and SNI–alcohol groups of animals

	Sham–water (*N* = 6, *n *= 16)	SNI–water (*N* = 5, *n* = 19)	Sham–alcohol (*N* = 7, *n* = 22)	SNI–alcohol (*N* = 6, *n* = 22)	Statistical data
Subthreshold properties					
Resting membrane potential (mV)	−76.71 ± 1.458	−78.11 ± 1.334	−75.98 ± 1.496	−72.45 ± 1.386	Surgery *p* = 0.465 Fluid *p = 0.030 Interaction *p* = 0.093
Input resistance (mΩ)	121.3 ± 5.862	126.5 ± 6.725	123.9 ± 5.479	139.9 ± 9.134	Surgery *p* = 0.147 Fluid *p* = 0.271 Interaction *p* = 0.454
Firing properties					
Threshold potential (mV)	−40.79 ± 1.053	−44.11 ± 0.7821	−42.12 ± 1.048	−40.65 ± 1.063	Surgery *p* = 0.365 Fluid *p* = 0.301 Interaction *p = 0.021
Rheobase (pA)	181.3 ± 11.06	184.2 ± 12.71	170.5 ± 13.43	159.1 ± 13.42	Surgery *p* = 0.750 Fluid *p* = 0.176 Interaction *p* = 0.587
Number of action potentials at 400 pA	15.75 ± 0.5515	15.89 ± 0.8017	14.91 ± 0.9735	19.82 ± 1.205	Surgery *p = 0.012 Fluid *p* = 0.120 Interaction *p = 0.018
Height (mV)	76.52 ± 1.331	78.01 ± 1.668	82.64 ± 1.930	79.07 ± 2.619	Surgery *p* = 0.617 Fluid *p* = 0.087 Interaction *p* = 0.227
FI slope (Hz/pA)	0.1271 ± 0.005	0.1265 ± 0.005	0.1161 ± 0.005	0.1436 ± 0.006	Surgery *p = 0.018 Fluid *p* = 0.584 Interaction *p = 0.014
Fast after hyperpolarization (mV)	−7.470 ± 1.452	−7.718 ± 1.094	−6.879 ± 1.127	−6.266 ± 0.8855	Surgery *p* = 0.872 Fluid *p* = 0.369 Interaction *p* = 0.704
Spike frequency adaptation ratio (3rd/5th)	0.9848 ± 0.01806	0.8985 ± 0.03885	0.8769 ± 0.02320	0.9599 ± 0.02428	Surgery *p* = 0.951 Fluid *p* = 0.403 Interaction **p = 0.003

Two-way ANOVA; data shown as mean ± standard error of the mean. *p < 0.05; **p < 0.01.

### The combination of SNI and alcohol consumption increases the frequency of mEPSCs of AIC→DLS neurons

**Figure 7.**
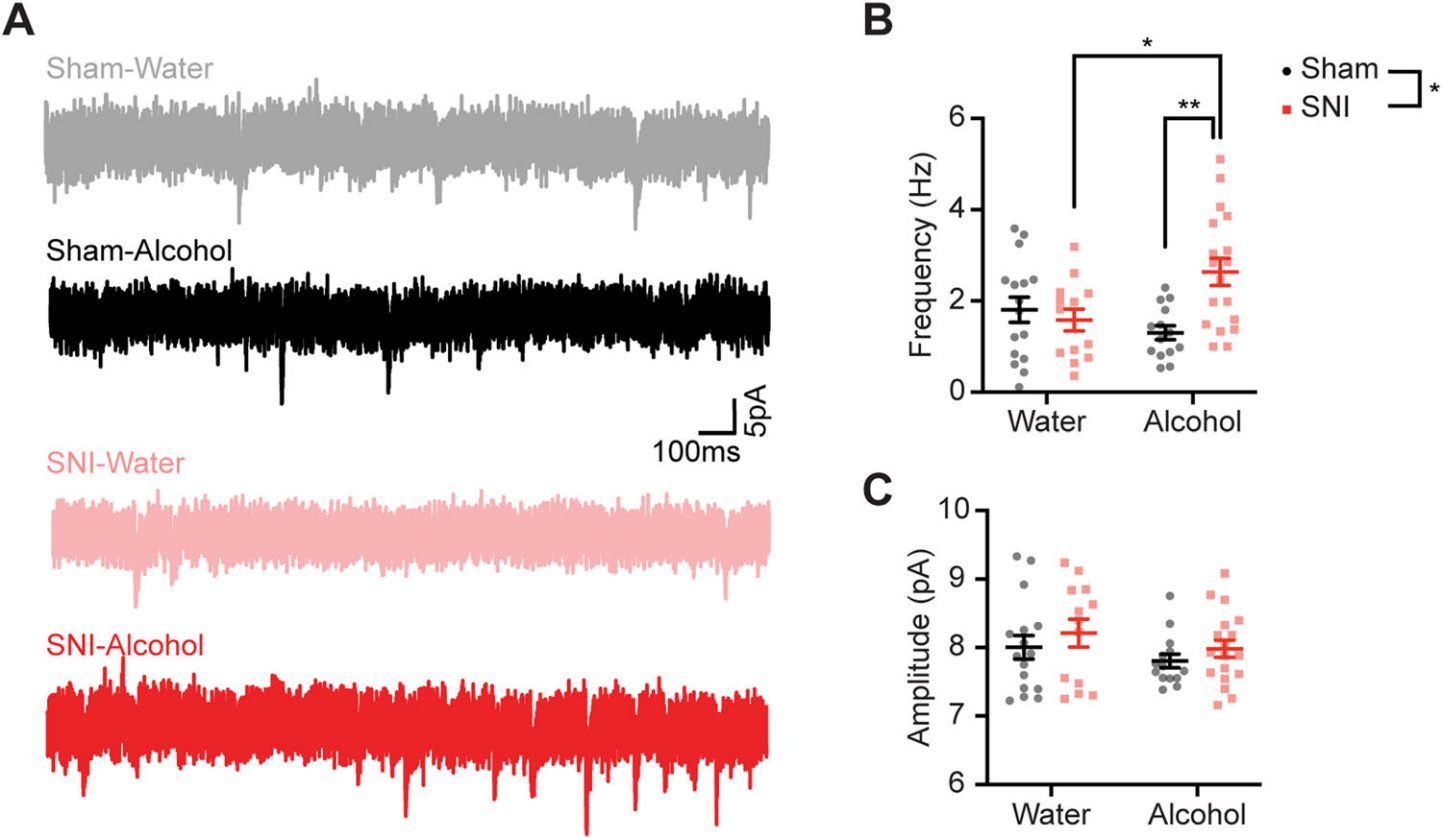
The combination of SNI and alcohol consumption increases the mEPSC frequency of AIC→DLS neurons. ***A***, Example traces of mEPSCs from AIC→DLS neurons from sham–water (gray, *N* = 6, *n* = 16), sham–alcohol (black, *N* = 5, *n* = 13), SNI–water (orange, *N* = 6, *n* = 14), and SNI–alcohol (red, *N* = 6, *n* = 18) mice. ***B***, Neurons from SNI–alcohol mice displayed an increased frequency of mEPSCs compared with those from the sham–alcohol and SNI–water groups. ***C***, No difference observed in the amplitude of mEPSCs among all four groups. *p < 0.05. **p < 0.01. *N*, animal number; *n*, cell number.

We next tested for differences in excitatory synaptic transmission onto AIC→DLS neurons by recording mEPSCs in the AIC across the four experimental groups (Fig. 7*A*). A significant increase in mEPSC frequency was detected in AIC→DLS neurons from SNI–alcohol mice compared with that from SNI–water and sham–alcohol groups (Fig. 7*B*; two-way ANOVA: fluid, *F*_(1,57)_ = 1.097, *p* = 0.299; surgery, *F*_(1,57)_ = 4.443, *p* = 0.039; interaction, *F*_(1,57)_ = 8.822, *p* = 0.004; Tukey's test: SNI–water vs SNI–alcohol, *p* = 0.03; sham–alcohol vs SNI–alcohol, *p* = 0.003). No statistical differences were observed in the amplitude of mEPSCs (Fig. 7*C*; two-way ANOVA: fluid, *F*_(1,57)_ = 1.936, *p* = 0.170; surgery, *F*_(1,57)_ = 1.574, *p* = 0.215; interaction, *F*_(1,57)_ = 0.009343, *p* = 0.923). Increased mEPSC frequency without a change in mEPSC amplitude indicates that SNI plus alcohol consumption enhances presynaptic glutamate release onto AIC→DLS neurons. It is likely that enhanced presynaptic release of the glutamate is a contributing factor in the increased excitability of AIC→DLS neurons recorded from SNI–alcohol mice.

### AIC→DLS neuronal excitability is not significantly altered in male mice after 1 week of DID

**Figure 8.**
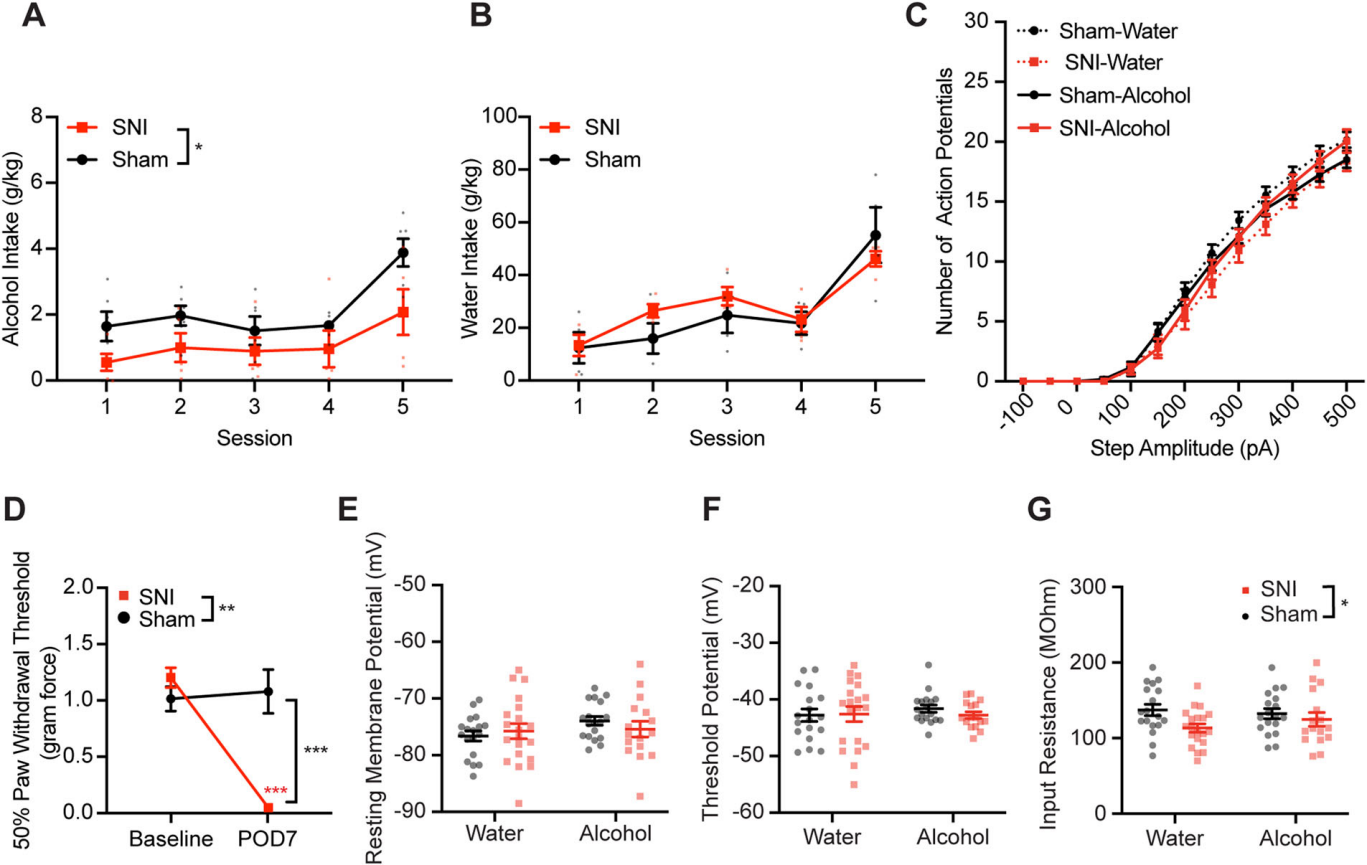
The combination of SNI and alcohol has no significant effect on the AIC→DLS neuronal excitability after 1 week DID. ***A***, SNI mice had significantly lower alcohol intake compared with sham mice (sham, *N* = 6; SNI, *N* = 5). ***B***, SNI and sham mice had similar water intake during 1 week DID (sham, *N* = 4; SNI, *N* = 4). ***C***, All four groups of animals showed similar action potential firing frequency of AIC→DLS neurons in response to depolarizing current steps. ***D***, SNI mice showed significantly lower PWT on POD7 compared with baseline. ***E***, There is no difference in resting membrane potential among the four groups. ***F***, There is no difference in threshold potential among the four groups. ***G***, SNI mice show significantly lower input resistance compared with sham group mice (sham–water, *N* = 4, *n* = 18; SNI–water, *N* = 4, *n* = 20; sham–alcohol, *N* = 5, *n* = 18; SNI–alcohol, *N* = 4, *n* = 16). *p < 0.05. **p < 0.01. ***p < 0.001. *N*, animal number; *n*, cell number.

We next determined if intrinsic properties of AIC→DLS neurons are altered earlier in the DID paradigm. Therefore, we performed electrophysiology recordings on SNI and sham male mice after only 1 week of DID. Consistent with the 3 week DID findings, SNI mice drank significantly less alcohol compared with sham mice during this 1 week DID (Fig. 8*A*; rmANOVA: surgery, *F*_(1,9)_ = 5.421, *p* = 0.045; time, *F*_(2.867,25.80)_ = 11.83, *p* < 0.001; interaction, *F*_(4,36)_ = 1.084, *p* = 0.379). SNI and sham mice consumed similar amount of water throughout the week (Fig. 8*B*; rmANOVA: surgery, *F*_(1,6)_ = 0.1683, *p* = 0.696; time, *F*_(2.111,12.67)_ = 20.37, *p* < 0.001; interaction, *F*_(4,24)_ = 1.381, *p* = 0.270). SNI mice showed mechanical allodynia 7 d after surgery (Fig. 8*D*; rmANOVA: surgery, *F*_(1,17)_ = 10.15, *p* = 0.005; time, *F*_(1,17)_ = 21.76, *p* = 0.001; interaction, *F*_(1,17)_ = 27.28, *p* < 0.001; Sidak's post hoc test: SNI baseline vs POD7, *p* < 0.001; POD7 SNI vs sham, *p* < 0.001). Our recording analyses revealed no significance effects of fluid, surgery, or fluid–surgery interaction on action potential firing (Fig. 8*C*; rmANOVA: current, *F*_(12,816)_ = 1029, *p* < 0.001; fluid, *F*_(1,68)_ = 0.001248, *p* = 0.972; surgery, *F*_(1,68)_ = 2.135, *p* = 0.149; current × fluid, *F*_(12,816)_ = 0.07712, *p* > 0.999; current × surgery, *F*_(12,816)_ = 2.013, *p* = 0.021; fluid × surgery, *F*_(1,68)_ = 2.258, *p* = 0.138; current × fluid × surgery, *F*_(12,816)_ = 2.427, *p* = 0.004). In addition, no significant difference was detected in resting membrane potential or threshold potential across the four experimental groups (Fig. 8*E*,*F*). However, there is a main surgery difference in input resistance, indicating that SNI mice had significantly lower input resistance than sham mice, regardless of fluid intake (Fig. 8*G*; two-way ANOVA: fluid, *F*_(1,68)_ = 0.2036, *p* = 0.653; surgery, *F*_(1,68)_ = 4.824, *p* = 0.031; interaction, *F*_(1,68)_ = 1.328, *p* = 0.253). Overall, these data indicate that AIC→DLS neuronal excitability is unchanged by SNI and only 1 week of alcohol DID.

### Alcohol exposure prior to SNI ablates the differences in DID alcohol intake and AIC→DLS neuronal excitability observed in mice with no alcohol pre-exposure prior to surgery

**Figure 9.**
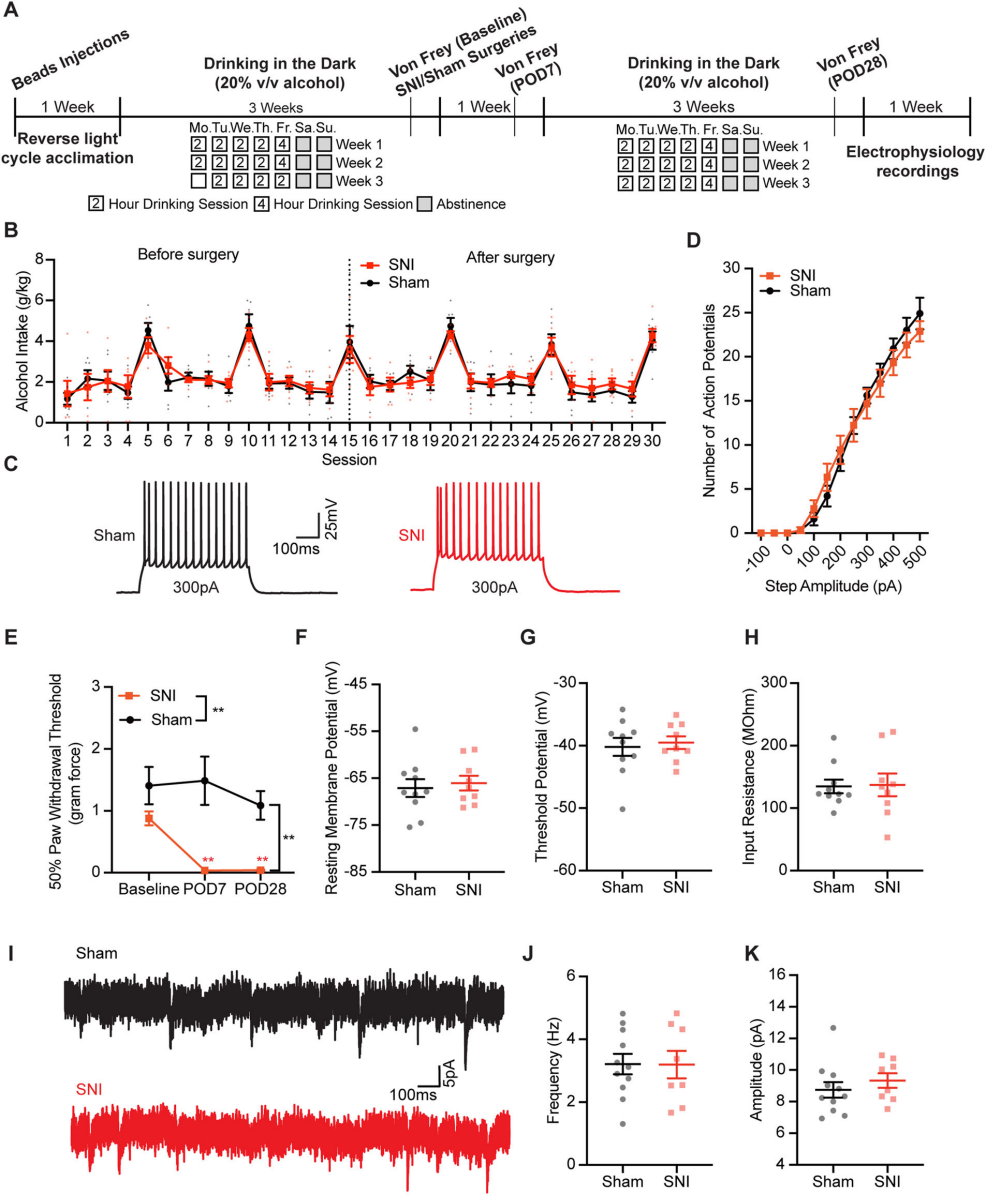
Alcohol pre-exposure has no effect on alcohol intake and AIC→DLS neurons excitability after injury. ***A***, Schematic of the experimental timeline. ***B***, No difference in alcohol intake after injury between sham and SNI animals (sham, *N* = 6; SNI *N* = 6). ***C***, Example traces of current-evoked action potential firing from AIC→DLS neurons from sham (black) and SNI (red) animals. ***D***, Frequency–input (FI) current curves showing that the frequency of action potential firing of AIC→DLS neurons in response to depolarizing current steps in sham mice (*N* = 4, *n* = 10) has no difference from cells in SNI mice (*N* = 5, *n* = 9). ***E***, Mechanical allodynia was observed on POD7 after SNI of the sciatic nerve and persisted through POD 28 (sham, *N* = 6; SNI, *N* = 6). No difference of AIC→DLS neurons was observed between sham and SNI mice in (***F***) resting membrane potential, (***G***) threshold potential, and (***H***) input resistance. ***I***, Example traces of mPESCs from AIC→DLS neurons from sham (black) and SNI (red) mice. There is no difference in mEPSC (***J***) frequency and (***K***) amplitude between sham (*N* = 3, *n* = 11) and SNI (*N* = 3, *n* = 8) mice. **p < 0.01. *N*, animal number; n, cell number.

Pre-existing substance use disorders contribute to higher levels of pain after injury (Turk, 1997), which may play a role in the continuation of the alcohol drinking problems (Brennan et al., 2005). Individuals with current alcohol drinking problems are more prone to use alcohol as a coping method including for pain (Moos, 1990; Wunschel et al., 1993; Moggi et al., 1999; Brennan et al., 2005). In our initial experimental design, SNI mice did not drink alcohol prior to SNI surgery. Therefore, we exposed mice to alcohol via 3 weeks of DID prior to SNI/sham surgery (Fig. 9*A*). Animals that had SNI surgery showed significantly decreased PWT on POD7 and POD28 compared with baseline (Fig. 9*E*; rmANOVA: surgery, *F*_(1,10)_ = 13.83, *p* = 0.004; time, *F*_(2,20)_ = 7.739, *p* = 0.003; interaction, *F*_(2,20)_ = 4.751, *p* = 0.021; Sidak's post hoc test: POD7 SNI vs sham, *p* < 0.001; POD28 SNI vs sham, *p* = 0.003; SNI baseline vs POD7, *p* = 0.002; SNI baseline vs POD28, *p* = 0.002; SNI POD7 vs POD28, *p* > 0.999). When pre-exposed to alcohol, SNI and sham mice consumed similar amounts of alcohol in the 3 weeks of DID after injury (Fig. 9*B*; Sessions 16–30, rmANOVA: surgery, *F*_(1,10)_ = 0.06040, *p* = 0.811; time, *F*_(4.486,44.86)_ = 25.81, *p* < 0.001; interaction, *F*_(14,140)_ = 0.6392, *p* = 0.828). Furthermore, no significant difference was detected in the number of action potentials recorded from AIC→DLS neurons between sham and SNI mice pre-exposed to alcohol (Fig. 9*C*,*D*; rmANOVA: surgery, *F*_(1,17)_ = 0.03350, *p* = 0.857; current, *F*_(2.242,38.12)_ = 279.6, *p* < 0.001; interaction, *F*_(12,204)_ = 1.279, *p* = 0.233). Resting membrane potential, action potential threshold potential, and input resistance were also not significantly different between AIC→DLS neurons recorded from sham and SNI animals (Fig. 9*F–H*; two-tailed unpaired *t* test; resting membrane potential, *p* = 0.673; threshold potential, *p* = 0.704; input resistance, *p* = 0.906). Lastly, mEPSC analysis showed that AIC→DLS neurons from sham and SNI mice had similar mEPSC frequency and amplitude (Fig. 9*I–K*; two-tailed unpaired *t* test; frequency, *p* = 0.974; amplitude, *p* = 0.408).

**Figure 10.**
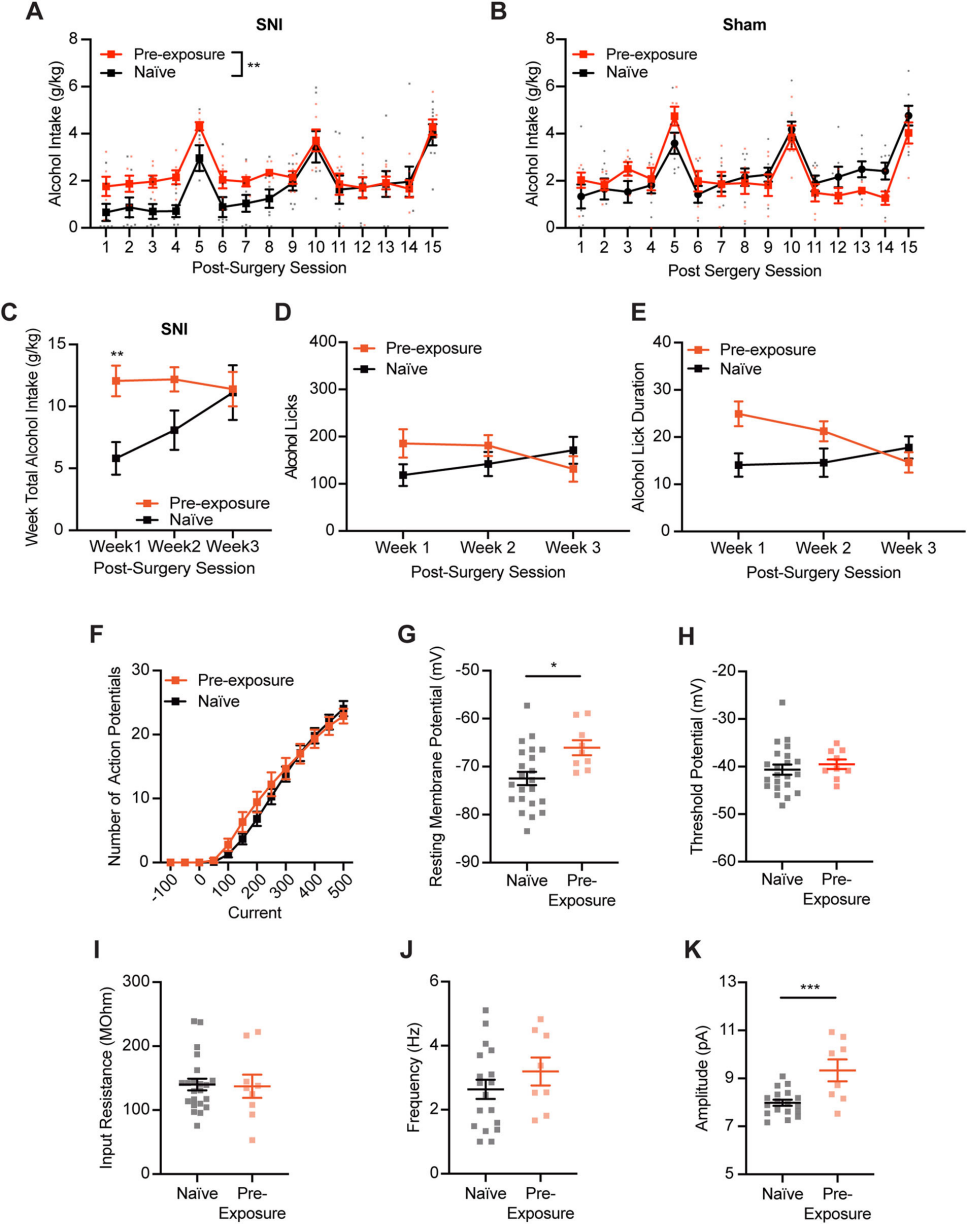
Comparison between SNI mice with and without alcohol pre-exposure. ***A***, ***C***, SNI mice with alcohol pre-exposure had higher alcohol intake after surgery compared with mice naive to alcohol (***A***, by day; ***C***, by week). ***B***, Comparison of alcohol drinking between naive and pre-exposed sham mice displayed no significant difference (naive, *N* = 8; pre-exposure, *N* = 6). Significant interaction between alcohol pre-exposure and postsurgery drinking session observed in (***D***) alcohol licks and (***E***) alcohol lick duration (naive, *n* = 8; pre-exposure, *n* = 6). ***F***, No difference detected in the number of action potential firing in response to depolarizing current steps between naive and pre-exposed mice (naive, *N* = 6, *n* = 22; pre-exposure, *N* = 5, *n* = 9). ***G***, SNI mice with alcohol pre-exposure had significantly depolarized resting membrane potential compared with SNI mice naive to alcohol prior to injury. There is no difference in (***H***) threshold potential, (***I***) input resistance, and (***J***) mEPSC frequency between mice with and without alcohol pre-exposure. ***K***, Animals pre-exposed to alcohol had significantly increased amplitude of mEPSCs compared with animals naive to alcohol before surgery (naive, *N* = 6, *n* = 18; pre-exposure *N* = 3, *n* = 8). *p < 0.05. **p < 0.01. ***p < 0.001. *N*, animal number; *n*, cell number.

Compared with mice naive to alcohol (mice with no alcohol pre-exposure prior to surgery), we found that SNI animals with alcohol pre-exposure had higher alcohol intake after surgery (Fig. 10*A*,*C*; rmANOVA, by day: pre-exposure, *F*_(1,12)_ = 15.8540, *p* = 0.0026; postsurgery session, *F*_(14,140)_ = 15.5040, *p* < 0.001; interaction, *F*_(14,140)_ = 0.9368, *p* = 0.5215; by week: pre-exposure, *F*_(1,12)_ = 3.4415, *p* = 0.0883; postsurgery week, *F*_(2,24)_ = 3.0636, *p* = 0.0653; interaction, *F*_(2,24)_ = 3.6520, *p* = 0.0412; Sidak's post hoc test: week 1 naive vs pre-exposed, *p* = 0.0050). Analyses of drinking microstructure data collected from lickometers identified a significant interaction between alcohol pre-exposure and DID drinking session with increased number of total licks and lick duration observed in weeks 1 and 2 (Fig. 10*D–E*; rmANOVA, total licks: pre-exposure, *F*_(1,12)_ = 0.1847, *p* = 0.6750; postsurgery session, *F*_(2,24)_ = 0.0687, *p* = 0.9338; interaction, *F*_(2,24)_ = 3.9202, *p* = 0.0336; lick duration: pre-exposure, *F*_(1,12)_ = 0.9828, *p* = 0.3411; postsurgery session, *F*_(2,24)_ = 0.8791, *p* = 0.4281; interaction, *F*_(2,24)_ = 5.6566, *p* = 0.0097). Alcohol intake between sham control animals in naive and pre-exposed groups was not statistically different, indicating that these two independent experiments are comparable (Fig. 10*B*; rmANOVA: pre-exposure, *F*_(1,12)_ = 0.04422, *p* = 0.837; session, *F*_(4.637,54.98)_ = 24.54, *p* < 0.001; interaction, *F*_(14,166)_ = 3.097, *p* < 0.001). Electrophysiology recording comparisons of AIC→DLS neurons showed no difference in the number of action potential firing between SNI-naive and SNI pre-exposure mice (Fig. 10*F*; rmANOVA: pre-exposure, *F*_(1,29)_ = 0.2034, *p* = 0.655; current, *F*_(1.501,43.52)_ = 280.7, *p* < 0.001; interaction, *F*_(12,348)_ = 1.338, *p* = 0.195). Threshold potential for action potential firing, input resistance, and mEPSCs frequency were also not significantly different between the two groups (Fig. 10*H–J*; two-tailed unpaired *t* test; threshold potential, *p* = 0.530; input resistance, *p* = 0.885; mEPSCs frequency, *p* = 0.307). However, SNI animals with alcohol pre-exposure had significantly depolarized resting membrane potential (Fig. 10*G*; two-tailed unpaired *t* tests; *p* = 0.012) and increased mEPSC amplitudes (Fig. 10*K*; two-tailed unpaired *t* tests; *p* < 0.001) compared with alcohol-naive mice. Overall, these data suggest that pre-exposure to alcohol before injury eliminates most of the disparities we detected in both alcohol consumption and AIC→DLS neuronal activity compared with DID-SNI mice with no prior exposure to alcohol.

## Discussion

Our results show that SNI mice with no prior exposure to alcohol (alcohol-naive) consume significantly less alcohol in the DID paradigm compared with sham mice. AIC→DLS neurons recorded from these same SNI–alcohol mice display increased neuronal excitability compared with AIC→DLS neurons recorded from sham–alcohol, sham–water, and SNI–water groups. We provide evidence that the combination of SNI and alcohol-DID enhances presynaptic glutamatergic inputs onto AIC→DLS neurons recorded from alcohol-naive mice. Interestingly, exposing mice to 3 weeks of alcohol DID prior to SNI surgery eliminated differences in both DID alcohol consumption and AIC→DLS neuronal activity observed in alcohol-naive mice.

We conclude that reduced alcohol intake observed in alcohol-naive SNI mice is not due to attenuation in alcohol reward as SNI–alcohol and sham–alcohol mice both displayed intact alcohol CPP. Decreased saccharin drinking is known to be associated with anhedonia, which we considered as a potential contributing factor in decreased alcohol intake in alcohol-naive SNI mice (Willner, 1985; Willner et al., 1987; Harris et al., 1997; Crabbe et al., 2020). However, both SNI and sham animals drank similar amounts of saccharin, indicating that reduced alcohol intake of alcohol-naive SNI mice is not due to anhedonia. It is also unlikely that SNI blunted sensitivity to the sweet taste or rewarding value of saccharin as similar levels of intake were measured across groups (Willner et al., 1987; Crabbe et al., 2020). Another explanation for why alcohol-naive SNI mice consumed less alcohol is that the acute physiological effects of alcohol induce a greater pain response in SNI mice. Specifically, in SNI mice, low doses of alcohol could induce a pain phenotype that discourages mice from escalating their intakes, leading to drinking cessation earlier in DID sessions compared with sham controls. Unfortunately, testing mechanical allodynia shortly after mice started DID is difficult to assess without altering future intakes during the same DID session. Further, recording any difference between SNI–alcohol and SNI–water mice may be challenging since both groups reached a near floor effect in von Frey response at and beyond POD7.

The DID experiments showing that alcohol-naive SNI mice drank less alcohol compared with sham control support our null hypothesis and do not align with clinical findings that patients with chronic pain are more likely to drink alcohol (Brennan et al., 2005; Riley and King, 2009; Zale et al., 2015). Most likely, these patients have had exposure to alcohol prior to the development of chronic pain. Therefore, we exposed mice to 3 weeks of alcohol-DID prior to initiating the SNI and found that SNI–alcohol and sham–alcohol mice drank similar levels of alcohol in the 3 weeks of DID following surgery. These data clearly show that SNI mice with prior alcohol exposure binge drink alcohol differently than SNI mice without prior alcohol exposure. However, we did find that SNI mice with prior alcohol exposure did not binge drink significantly more alcohol than uninjured mice, which is not consistent with clinical data (Brennan et al., 2005; Riley and King, 2009; Zale et al., 2015). One potential explanation for this disparity is that the DID model is not revealing alcohol seeking behavior, which may be more pronounced in SNI mice. This will be a focus of future studies on alcohol and SNI.

Our electrophysiology data from SNI–alcohol mice showed that AIC→DLS neurons displayed aspects of increased intrinsic excitability. Specifically, alcohol depolarized the resting membrane potential of AIC→DLS neurons, which is consistent with work from our lab showing that binge alcohol consumption drives enhanced function of AIC inputs in the DLS (Haggerty et al., 2022). However, only the combination of SNI and alcohol drinking created an increased frequency of action potential firing, indicating that alcohol exposure alone does not enhance excitability of AIC→DLS neurons at the soma level. Further, we identified a significantly higher frequency, but not amplitude, of mEPSCs in AIC→DLS neurons from SNI–alcohol mice, indicating an enhanced presynaptic release of glutamate. One potential source of increased glutamatergic input onto AIC neurons is the basolateral amygdala (BLA). Glutamatergic BLA→AIC circuitry is involved in the maintenance of contextual memories associated with drug reward (Gil-Lievana et al., 2020). Moreover, the BLA processes ascending pain signals (Gauriau and Bernard, 2004; Neugebauer et al., 2004; Apkarian et al., 2013). Previous studies show that SNI alters BLA input onto prelimbic cortex (Huang et al., 2019; Cheriyan and Sheets, 2020). However, whether SNI and alcohol consumption alters BLA inputs onto AIC neurons remains to be determined. Anxiety is another factor known to regulate both alcohol drinking and chronic pain (Wilson, 1988; Bandelow, 2015; Chen et al., 2022). AIC mediates anxiety-related behaviors as clinical data suggested a positive correlation between self-reported measures of anxiety and the activity in the right AIC (Terasawa et al., 2013). In rodents, both AIC and the AIC→BLA pathway are indicated as neural substrates influencing emotional valence and anxiety (Nicolas et al., 2023). As such, future studies will focus on dissecting effects of DID and SNI on BLA→AIC→DLS circuits.

Intrinsic and synaptic hyperexcitability was only observed in AIC→DLS neurons recorded from SNI–alcohol mice with no prior exposure to alcohol. We have shown that optogenetically driving AIC→DLS transmission decreases binge alcohol consumption behaviors in mice (Haggerty et al., 2022). Together, this suggests that enhanced AIC→DLS activity driven by SNI and alcohol interaction is a contributing factor in our observed decrease in DID–alcohol intake in alcohol-naive mice. One possible explanation for this is that SNI induces a protective taste neophobia that has been observed in rats following surgery (Bauer et al., 2003). Previous studies demonstrate that memory for experiencing a new taste is dependent on the functionality of the insular cortex (Gal-Ben-Ari and Rosenblum, 2011; Yiannakas and Rosenblum, 2017). Furthermore, hyperexcitability of neurons in deeper layers (i.e., L5/6) of AIC is implicated in taste neophobia (Kayyal et al., 2021). While this study identified these AIC neurons as mPFC projecting, it is conceivable that these same neurons send projections to DLS. Based on these findings and our current data, it is possible that SNI hypersensitizes AIC→DLS neurons to the novel and bitter taste of alcohol, which leads to a pronounced taste aversion/neophobia and decreased alcohol consumption in alcohol-naive mice. SNI animals with alcohol pre-exposure drank significantly more alcohol during the first week of DID after surgery compared with alcohol-naive SNI animals. Interestingly, significant differences in the intrinsic excitability observed in SNI–alcohol mice without prior alcohol exposure were undetectable in SNI–alcohol mice with prior alcohol exposure. This suggests that pre-exposure to alcohol eliminates the novelty of alcohol taste following SNI, thereby attenuating both sensitization of AIC→DLS neurons and reduction in alcohol-DID. Surprisingly, we did not observe increased excitability of AIC→DLS neurons in SNI mice following 1 week of alcohol-DID despite a reduction in alcohol consumption. This suggests that increased excitability of AIC→DLS neurons is driven by plasticity occurring later in the progression of alcohol-DID. Another possibility is that enhanced activity of AIC→DLS neurons in 3 week alcohol-DID SNI mice is associated with a compensatory mechanism that contributes to SNI mice eventually reaching similar alcohol consumption as sham at later stages of DID.

There are also several limitations of this study that need to be further addressed. More work need to be done in female mice, which displayed no changes to DID–alcohol drinking after SNI. Our focus on male mice for this study was made because our previous studies of alcohol effects on AIC→DLS circuitry revealed male-specific effects of alcohol (Haggerty et al., 2022). Yet, it is known that both SNI and binge alcohol consumption on their own produce outcomes with sex differences (Sneddon et al., 2019; Ahlström et al., 2021). Together, the interactions between sex, pain, alcohol, and the associated circuit and behavioral changes that they drive are of great interest to our future work and public health outcomes.

Future studies should investigate whether SNI and alcohol exposure produce the same outcomes for ipsilateral AIC→DLS neurons. In addition, AIC neurons also send projections to the amygdala (Gehrlach et al., 2020; Nicolas et al., 2023), meaning collateralization of AIC→DLS circuits could be examined. A recent study demonstrated that AIC→BLA neurons have collateral projections to ipsilateral nuclei including central amygdala and nucleus accumbens but predominantly to contralateral BLA (Nicolas et al., 2023). A comparable pattern might exist for AIC→DLS neurons and needs to be studied. Future electrophysiology recordings in other neuronal pathways need to be performed to determine whether SNI–alcohol interaction drives synaptic and intrinsic changes primarily to the AIC→DLS circuit. One study showed that inhibitory neurons in AIC display similar connectivity patterns and strength compared with pyramidal neurons (Gehrlach et al., 2020), which indicates that the balance of excitation and inhibition in the AIC should be studied with the combination of SNI and alcohol drinking.

In conclusion, we demonstrate that SNI reduces binge alcohol consumption and that the combination of SNI and alcohol drinking together for 3 weeks significantly increases somatic excitability and excitatory synaptic transmission in AIC→DLS circuits. How and whether a hyperexcitable state of the AIC→DLS pathway contributes to altered alcohol drinking behaviors in a neuropathic pain state will be the focus of future studies.
